# Ebolavirus is evolving but not changing: No evidence for functional change in EBOV from 1976 to the 2014 outbreak

**DOI:** 10.1016/j.virol.2015.03.029

**Published:** 2015-08

**Authors:** Abayomi S. Olabode, Xiaowei Jiang, David L. Robertson, Simon C. Lovell

**Affiliations:** a Computational and Evolutionary Biology, Faculty of Life Sciences, University of Manchester, Manchester, UK; b Department of Genetics, University of Cambridge, Cambridge, UK

**Keywords:** Ebola, Evolution, Adaptation, Protein structure, Protein function

## Abstract

The 2014 epidemic of Ebola virus disease (EVD) has had a devastating impact in West Africa. Sequencing of ebolavirus (EBOV) from infected individuals has revealed extensive genetic variation, leading to speculation that the virus may be adapting to humans, accounting for the scale of the 2014 outbreak. We computationally analyze the variation associated with all EVD outbreaks, and find none of the amino acid replacements lead to identifiable functional changes. These changes have minimal effect on protein structure, being neither stabilizing nor destabilizing, are not found in regions of the proteins associated with known functions and tend to cluster in poorly constrained regions of proteins, specifically intrinsically disordered regions. We find no evidence that the difference between the current and previous outbreaks is due to evolutionary changes associated with transmission to humans. Instead, epidemiological factors are likely to be responsible for the unprecedented spread of EVD.

## Highlights

• We study the current and previous outbreaks of Ebola virus disease.
• There are many non-synonymous (amino-acid altering) changes in the viral sequences.
• We can identify no changes that alter any molecular functions in the virus.
• The large number of infections is probably due to epidemiological factors.

## Introduction

The current ebolavirus epidemic in West Africa is characterized by an unprecedented number of infections, and has resulted in nearly 10,000 fatalities to date. A recent study using whole-genome sequencing methods (Gire et al., 2014) identified high levels of variation in the ebolavirus (EBOV), much of it unique to the current outbreak. Specifically, 341 substitutions were found of which 35 are non-synonymous nucleotide changes, *i.e.*, amino-acid residue altering. These changes, coupled with those that occurred prior to the current outbreak, indicate that the virus is evolving rapidly within humans. This presence of the non-synonymous substitutions in the EBOV genomes has led to concern that functional adaptation of the virus to the human host has already occurred (Gire et al., 2014; Basler 2014), accounting for the unusual scale and severity of the current outbreak.

According to the neutral theory of molecular evolution (Kimura 1983; Ohta 1973) most evolutionary changes at the molecular level are influenced by genetic drift. Mutations will be subject to positive selection if they are beneficial, purifying selection if they are deleterious, or evolve neutrally if their functional effect is neutral or nearly-neutral. Gire et al speculate that the amino acid replacements observed in the 2014–2015 EBOV population are due to incomplete purifying selection (Gire et al., 2014). If this is the case, we would expect replacements to be deleterious and their presence in the population to be due to selection having insufficient time to remove them. By contrast, positive selection may arise if any replacement increases viral fitness. This may be in the form of replacements that stabilize the protein structure, changes that have a beneficial effect on molecular function, or that permit the virus to escape the immune system. Neutral evolution would occur if replacements have minimal effect on fitness. We expect such replacements would be found predominantly in regions of the proteins that are not associated with defined functions, (*i.e.*, away from active sites and interaction sites with other molecules), have minimal effect on protein structure, either positive or negative, and not be in sites subject to selection from the immune system. Neutral evolution is the null hypothesis, and should be assumed in the absence of evidence for either positive or purifying selection.

We use the available EBOV sequence data to investigate whether there is evidence of deleterious, functional or adaptive changes in the viral genome. EBOV is a negative stranded RNA virus with a genome comprising seven genes. One of these genes (GP) gives rise to two protein products *via* transcriptional editing (Sanchez et al., 1996): a membrane-bound glycoprotein and a secreted protein. Additional functional diversity arises from VP40 existing in a number of conformational forms, which have different functions (Bornholdt et al., 2013). Protein structural data are available for at least part of six of the seven EBOV proteins, including three conformational forms for VP40 (Bornholdt et al., 2013; Gomis-Rüth et al., 2003; Hartlieb et al., 2007; Lee et al., 2008; Leung et al., 2009; Zhang et al., 2012; Dziubańska et al., 2014). These data allow us to investigate the structural and functional effects of amino acid replacements in the virus.

## Results

**Fig. 1:**
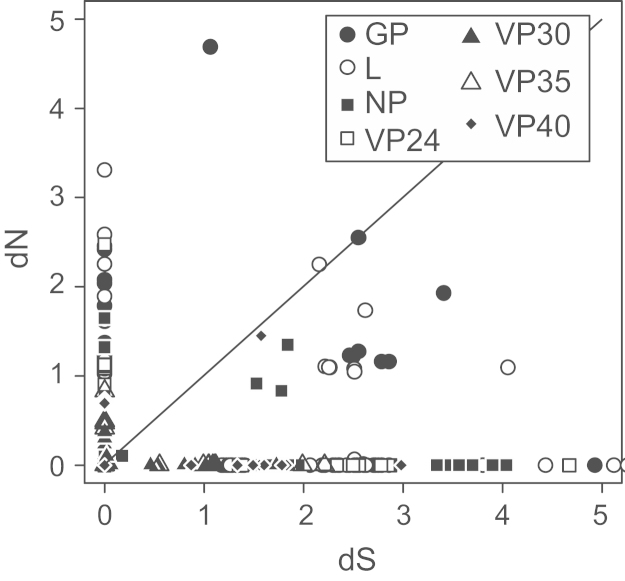
Number of synonymous (d*S*) and non-synonymous (d*N*) changes per site. Changes as calculated by the single-likelihood ancestor counting (SLAC) model (Kosakovsky Pond and Frost, 2005). The line indicates d*N*=d*S*.

We first investigated whether there is evidence for positive selection acting in any of the seven EBOV genes, using all of the available data (1976 to present). We calculated the ratio of non-synonymous (d*N*) changes to synonymous changes (d*S*, non-amino acid altering). A d*N*/d*S* ratio close to 1 indicates lack of selection, <1 purifying selection, and >1 that positive selection (adaptive evolution) has taken place. For all seven genes we find few sites with evidence for positive selection (Fig. 1 and Supplementary Table 1). However, except for one case, sites where d*N*/d*S*>1 have very low estimates of d*S*, suggesting that d*N*/d*S* ratios are unreliable in this context. For the exception, codon 430 in GP, the single-likelihood ancestor counting (SLAC) model estimates d*N*/d*S* to be 4.4. This replacement is not specific to the 2014 outbreak and in a region of the protein predicted to be intrinsically disordered. Disordered regions are known to be permissive for residue changes because they do not have well-defined structures and so are relatively unconstrained (Xue and Uversky, 2014), with higher evolutionary rates than non-disordered regions (Brown et al., 2011). There are also sites with d*N* but no d*S* values such that the d*N*/d*S* ratio cannot be calculated.

**Fig. 2:**
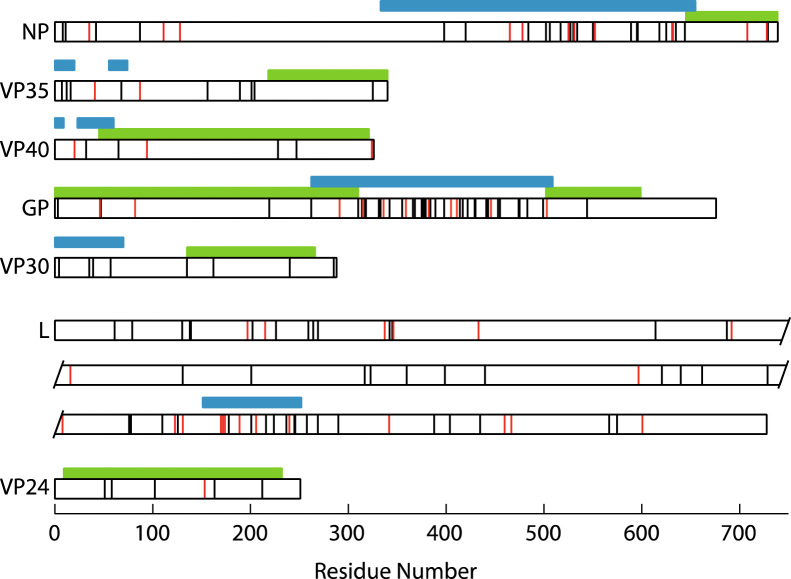
The distribution of amino acid replacements in the protein sequences. Proteins are shown as white horizontal bars, with replacements indicated by vertical lines. Replacements specific to the 2014 outbreak (Gire et al., 2014) are shown in red. Regions for which the protein structure has been determined are indicated in green, and those regions predicted to be disordered by Disopred (Ward et al., 2004) longer than 10 residues are indicated in blue. The polymerase (L) is broken across three lines for convenient representation.

To identify regions of enrichment for amino acid changes, we counted the number of d*N* replacements within windows of a range of sizes. Due to the relatively low number of changes, this analysis is somewhat sensitive to window size; values for a window size of 249 nucleotides (83 codons), incremented by 45 nucleotides, are shown in Supplementary Table 2. There is indication of enrichment towards the C-terminal end of VP30, although this due to only two non-synonymous changes in a region of low d*S*. Interestingly, we identify enrichment for d*N* in regions of proteins that are predicted to be intrinsically disordered. Altogether half of the amino acid replacements (89/177 for all sequences, 21/35 for the 2014 outbreak) are in disordered regions, despite these regions constituting only 27% of the protein sequence (Fig. 2). In the GP protein the central disordered section corresponds to a mucin-like protein which is highly glycosylated (Sanchez et al., 1996). Within disordered sequences such as mucin the general character of the amino-acid residue is important for function, but, glycosylation sites aside, specific interactions are not made, allowing a range of different amino acids to contribute to the same functional role (Nishikawa et al., 2010). Disordered regions have been implicated in the formation of new protein interactions (Meszaros et al., 2009) and may permit viruses to explore novel host perturbations *via* “sticky” interactions with host proteins (Xue and Uversky, 2014). In many cases these interactions are mediated through conserved short linear motif (SLiMs) (Davey et al., 2012) which may be located in disordered regions (Dosztanyi et al., 2009). Comparing predicted disorder for the various EBOV outbreak, we find there are no discernable differences (Fig. 4), indicating that although disordered regions are associated with amino acid changes there are no 2014-specific differences. When we predict binding regions within disordered regions based on disorder analysis, we find that of the 89 replacements in disordered regions, 46 are in regions that are predicted to be disordered (13 of which are 2014-specific). However, these replacements are predicted not to change the binding regions, with the exception of a small motif around residue 310 in GP, which is present in all outbreaks except for 1995 (Supplementary Figure 2).

**Fig. 3:**
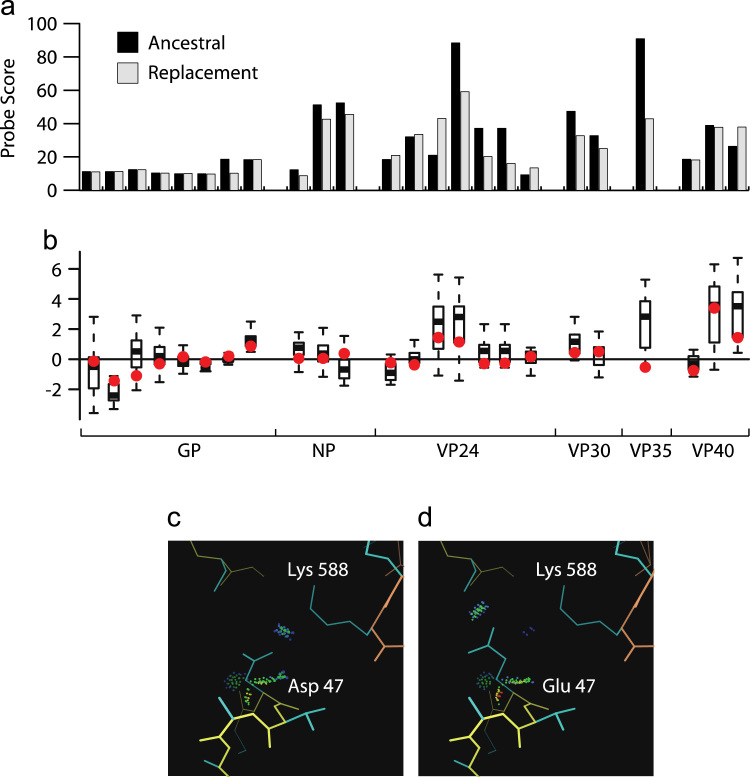
Analysis of amino acid replacements in the context of protein structure. (A) Probe score (Word et al., 1999) (arbitrary units /Å^2^×1000) for ancestral residues and replacements. Van der Waals overlaps, if present, would result in large negative scores, whereas favorable interactions result in positive scores. (B) Change in energy (ΔΔ*G*) for all amino acid replacements found in regions of known protein structure, as predicted using a statistical potential. Each boxplot represents a distribution of energy changes to all 19 other residue types at positions where a non-synonymous substitution has been observed. The ΔΔ*G* of the observed substitution is indicated in red. (C) Residue 47 of GP. GP1 is indicated by yellow main chain atoms and GP2 by orange. Van der Waals interactions between the side chain and surrounding atoms are shown by all-atom contact dots (Word et al., 1999); favorable interactions are colored blue, green and yellow; no unfavorable interactions are found. D. The replacement side chain, modeled in the tt0 rotamer (Lovell et al., 2000). Both the ancestral and replacement side chains are negatively charged, and are in close proximity to the positively charged lysine 588 of GP2.

As the d*N*/d*S* ratio is a relatively conservative method for detecting functional change we computationally characterized all individual amino acid replacements. For the amino acid replacements that fall within a region of known protein structure (Fig. 2) we assessed the likely functional effect of the change. To do this we used phylogenetic methods to reconstruct the most likely sequence of the most recent common ancestor and so the likely evolutionary trajectory. Firstly, we assess the goodness-of-fit of side chains in replacements in all proteins (Word et al., 1999). We find that all residue changes can be accommodated in the relevant protein structure in a low energy conformation (“rotamer”) with no substantial van der Waals overlaps (Fig. 3A). Secondly we used an empirical potential to predict the likely structural effect of a residue change (Guerois et al., 2002). For all replacements that can be assessed the ΔΔ*G* of folding associated with the amino acid replacement has a small magnitude compared to the background distribution (Fig. 3B) indicating that the likely effect on protein structure is minimal. We conclude that all replacements are compatible with their protein structure, and are unlikely to either increase or decrease protein stability.

For the GP, VP30 and VP40 proteins, the available protein structures allow identification of many of the protein–protein interaction interfaces *i.e.*, those regions of amino acid sequence responsible for virus to virus and virus to host binding. It should be noted that these structures contain many of the important interaction interfaces, but it is likely that both virus–virus and virus–host interactions are uncharacterized and so cannot be included in our analysis. In total 247 interface residues were identified (from all three proteins, including multiple VP40 conformation forms (Bornholdt et al., 2013)). Overwhelmingly, replacements in EBOV sequences, whether from the 2014 or earlier outbreaks, are found at sites that do not comprise interaction interfaces. The single exception is a replacement of aspartate 47 in GP1 to glutamate, which is not specific to the 2014 outbreak (Gire et al., 2014). Aspartate 47 makes an intra-chain salt bridge with lysine 588 in GP2. Structural modeling of this aspartate to glutamate replacement in GP1 indicates that there is a low energy conformation for glutamate that is able to make the same interaction with no van der Waals overlaps, suggesting this is a functionally conservative change (Fig. 3C). Note, the crystallographic temperature factors are high for this part of the protein structure (many are >100), indicating that the exact positioning of atoms is uncertain. Overall, we conclude that there is no evidence the identified protein interaction interfaces are being disrupted or otherwise altered by the observed amino acid replacements.

As structures are not available for all regions we next investigated the similarity of properties associated with the replacement residues. We find all are conservative for both hydropathy and volume, indicated by the small magnitude of the changes relative to the background distribution (see Supplementary Figure 1). Larger magnitude alterations are predominantly found in regions of the proteins that are predicted to be intrinsically disordered which are permissive for residue changes. These changes are therefore unlikely to affect structure or function.

A number of experimental studies have identified specific residues that are important for various functions. These include 18 residues in GP that are important for viral entry (Brindley et al., 2007; Manicassamy et al., 2005; Mpanju et al., 2006), five residues in VP30 that are required for nucleocapsid incorporation (Hartlieb et al., 2007), 23 residues in VP24 that are implicated in a range of protein–protein interactions (Zhang et al., 2012), four residues in VP40 that make direct contact with RNA (Gomis-Rüth et al., 2003) and a further two residues that are essential for budding (Bornholdt et al., 2013), and three residues in VP35 that are required for binding RNA (Leung et al., 2010). None of these residues are replaced in any of the EBOV lineages sequenced to date. We also examined “second shell” residues (those in contact with residues that play a direct role in function). Defining a residue contact using Probe we find that there are 116 second shell residues in total in these proteins. Only two of these are substituted. In GP, lysine 140 is implicated in viral entry to the cell (Brindley et al., 2007), and this is in contact with arginine 219 which is substituted to lysine. In VP24 leucine 119 has a suggested role in making protein–protein interactions (Zhang et al., 2012). This residue contacts isoleucine 153, which is substituted for valine. In both cases the replacements are conservative, and equivalent contacts can be made with the functional residues regardless of which of the alternative residues are present at the substituted site.

## Discussion

**Fig. 4:**
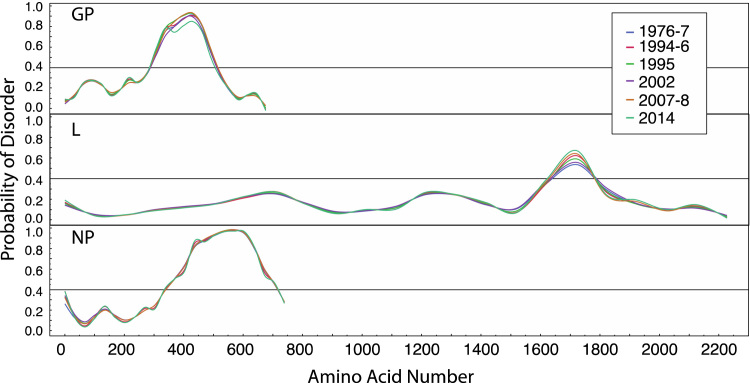
Prediction of disorder for GP, L and NP for sequences from each outbreak. Predictions were made with IUPred (Dosztanyi et al., 2005). The recommended cut off (0.4) for considering protein regions disordered is indicated.

Collectively, the structural and functional data indicate that the observed amino acid replacements are not found in regions of the protein that directly contribute to known functions, and, with the exception of GP aspartate 47, are not in any identified binding interface. None of the observed changes are likely to either stabilize or destabilize the protein structure. The non-synonymous changes are physicochemically conservative, and predominantly cluster in regions predicted to be intrinsically disordered. These changes are likely to be neutral as disordered regions are poorly constrained. Importantly, predictions of these disordered regions (Fig. 4) and potential binding sites within them indicate that the changes in these regions are not unique to the 2014 outbreak. Nonetheless given disordered regions potential role in the formation of new protein interactions (Meszaros et al., 2009; Xue and Uversky, 2014) such changes need to be monitored closely.

With the exception of a small number of low-frequency frame-shifting intrahost single nucleotide variants, we find no evidence for deleterious mutations that would have been consistent with incomplete purifying selection, as postulated by Gire et al. (2014). We also find no evidence of changes that are likely to be adaptive. This result is corroborated by analysis of EBOV from a phylogenetic perspective (Spielman et al., 2014) that similarly shows no evidence of positive selection in the 2014 outbreak. In addition we find no evidence of adaptive change in any of the EBOV sequences from past outbreaks. We conclude that none of the non-synonymous substitutions observed to date are likely to affect protein structure or function in any way, be it positive or negative. Thus the null hypothesis of neutral evolution cannot be rejected based on our analysis, and is the most reasonable explanation of the observed sequence diversity.

The reconstructed most likely genomes of the most recent common ancestor of each outbreak demonstrate the high degree of similarity of the progenitor virus of each outbreak. Coupled with a lack of functional change, this similarity points to a stable viral population in the animal reservoir, which is most likely to be fruit bats (Pigott et al., 2014). The zoonotic nature of EBOV and functional stability of the virus suggest that future transmission of similarly virulent potential is highly likely. Given the dense and highly connected nature of the human population, identification of the animal reservoir, surveillance and early intervention will be the key to prevention. The relative functional stability of EBOV suggests that intervention strategies (Pandey et al., 2014) such as with drugs or vaccinations may be more successful than for other RNA viruses.

It should be noted that functional change could be occurring due to factors that we have not analyzed. Changes may alter the function of the polymerase; this protein has only a short disordered region, and the structure is not known so it could not be subject to many of the analyses we have used. Changes in the intergenic regions (of which there are 14 identified in the 2014 outbreak) may be functional, and any changes may alter the efficiency of replication or translation, or RNA stability. However the 2014 outbreak is not more contagious than previous ones, has a similar rate of fatality and the clinical presentation is similar (Gatherer, 2015), indicating that any potential unidentified molecular changes are also likely to be neutral.

The lack of functional differences and any clinical distinction between the current and past Ebola outbreaks emphasizes the importance of human-centric epidemiological factors over the molecular biology and evolution of the virus (Gatherer, 2015). The main factor that differs between the 2014 outbreak and those that have occurred previously is the establishment of infections in relatively densely populated areas compared with previous outbreaks, coupled with poor health facilities (Gatherer, 2014). Human population growth and progressive urbanization have created efficient pathways for viral transmission (Pigott et al., 2014) despite a comparably low basic reproductive number, *R*_0_ (Gatherer, 2014).

Evolution of a change in mode of transmission of EBOV is extremely unlikely, due to the highly specific and intricate nature by which viruses interact with their hosts. Of more concern is the possibility that *R*_0_, the number of transmissions per infection, increases. Due to the unusually deadly nature of EBOV, a milder virus with even a substantial drop in virulence would still frequently result in deadly infections on an epidemic or pandemic scale. Despite no observable functional change between 1976 and mid-2014, future functional change is possible, emphasizing the need for continued monitoring of viral evolution.

## Methods

Ebolavirus sequences were obtained from GenBank, see Gire et al. (2014) for accession numbers. In total 101 sequences were used. Protein structural data was as follows: NP, PDB code 4QAZ (Dziubańska et al., 2014); VP35, PDB code 3FKE (Leung et al., 2009); GP, PDB code 3CSY (Lee et al., 2008); VP30, PDB code 2I8B (Hartlieb et al., 2007); VP24 PDB code 3VNE (Zhang et al., 2012). For VP40, multiple conformations are available, and all were assessed: PDB codes 4LDD, 4LDM, 4LDB (Bornholdt et al., 2013) and 1H2C (Gomis-Rüth et al., 2003).

The sequences were aligned and phylogenetic trees were estimated using the WAG substitution model (Whelan and Goldman, 2001), implemented in RAXML (Stamatakis, 2014). Ancestral sequences of EBOV proteins were reconstructed using maximum likelihood, implemented by FastML (Ashkenazy et al., 2012). Using ancestral reconstruction, the evolutionary pathway for every EBOV sequence in our data set was traced to the last common ancestor, and the sequence of every internal node was compared with that of its ancestor.

The energy change for all amino acid replacements that fall within regions of known structure was predicted using an empirical force field as implemented in FoldX software (version 3 Beta 6). Mutant structures were generated using the “build model” function in FoldX by mutating the native residue in the wild type with the 19 other possible amino acid residues for each position where a non-synonymous mutation was observed. Where the sequence of the native protein differed from that of the crystal structure, the structure was predicted using Modeller (Sali and Blundell, 1993).

Goodness-of-fit of replacement residues in the context of the protein structure was calculated using Probe (Word et al., 1999) after addition of hydrogen atoms with Reduce (Word et al., 1999). Probe was also used to identify residues in interaction interfaces. In each case all low energy conformations (“rotamers”) (Lovell et al., 2000) were assessed, and the rotamer with the best Probe score was used. For replacement valine 325 to isoleucine in VP35, we use the conformation of *χ*1=–45° *χ*2=–50°, which is not the bottom of the energy well, but is nevertheless a favorable side chain conformation. For both FoldX and Probe analyses residues were substituted individually. This is appropriate, as there are no examples where substituted residues are in contact.

The SLAC method as implemented by the Datamonkey webserver (Kosakovsky Pond and Frost, 2005) (http://www.datamonkey.org) was used to estimate d*N* (nonsynonymous) and d*S* (synonymous) rates for each protein. This method estimates the d*N* and d*S* rates for each codon site and compare the observed rates with null expectations based on the used nucleotide substitution model.

Residue volumes were taken from Zamyatnin (1972) and hydropathy scales from Kyte and Doolittle (1982). Disordered regions of proteins were predicted by DISOPRED (Ward et al., 2004) and IUPred (Dosztanyi et al., 2005). Predictions of binding regions (potential SLiMs) based on analysis of disorder using ANCHOR (Dosztanyi et al., 2009).
