# Supplementary material for: Ebolavirus is evolving but not changing: No evidence for functional change in EBOV from 1976 to the 2014 outbreak
Source: Virology. 2015 Aug;482:202–7. doi: 10.1016/j.virol.2015.03.029 (PMC4503884; doi:10.1016/j.virol.2015.03.029)
Supplement: Supplementary file 3 — Supplementary data [file mmc3.pdf]

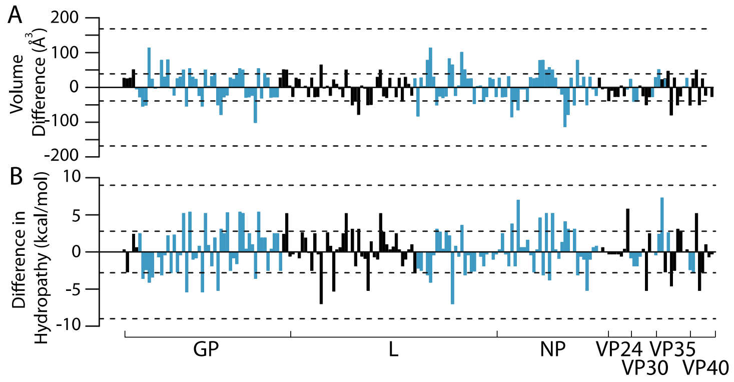

Supplementary Figure 1. Summary of changes of physicochemical properties arising from all amino acid replacements corresponding to non-synonymous substitutions occurring in EBOV between 1976 and 2014. (A) Volume change for each amino acid residue replacement. Upper and lower bounds, as well as the upper and lower quartiles are indicated by dashed lines. Replacements in regions that are predicted to be disordered are shown in blue, all others in black. (B) Change in hydropathy for each amino acid residue replacement. Upper and lower bounds, as well as the upper and lower quartiles are indicated by dashed lines. Replacements in regions that are predicted to be disordered are shown in blue, all others in black.

A.

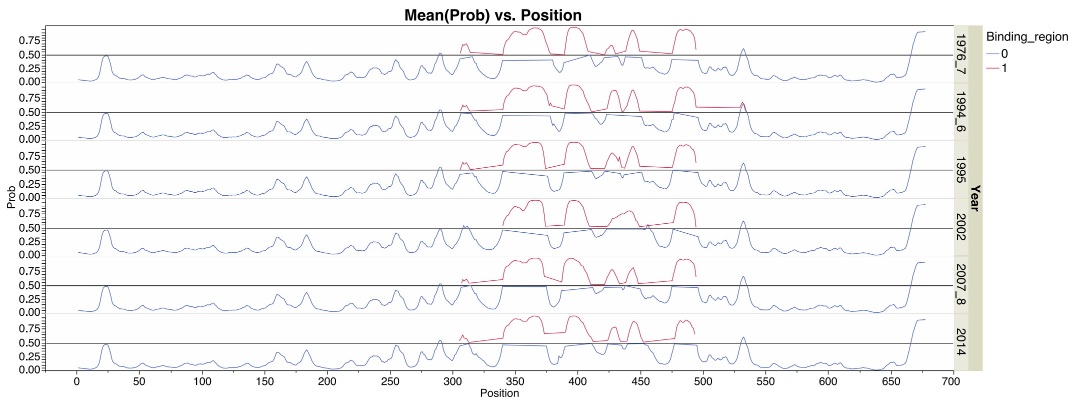

B.

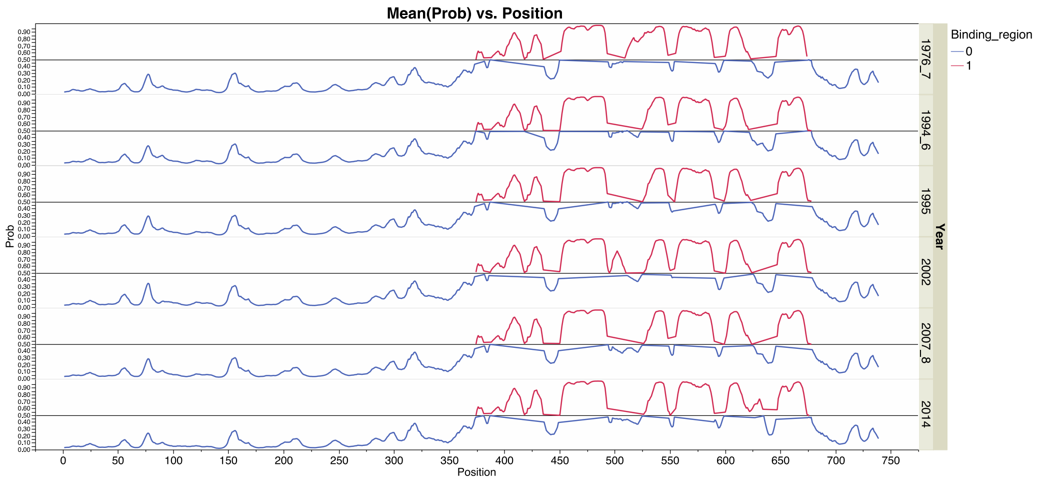

C.

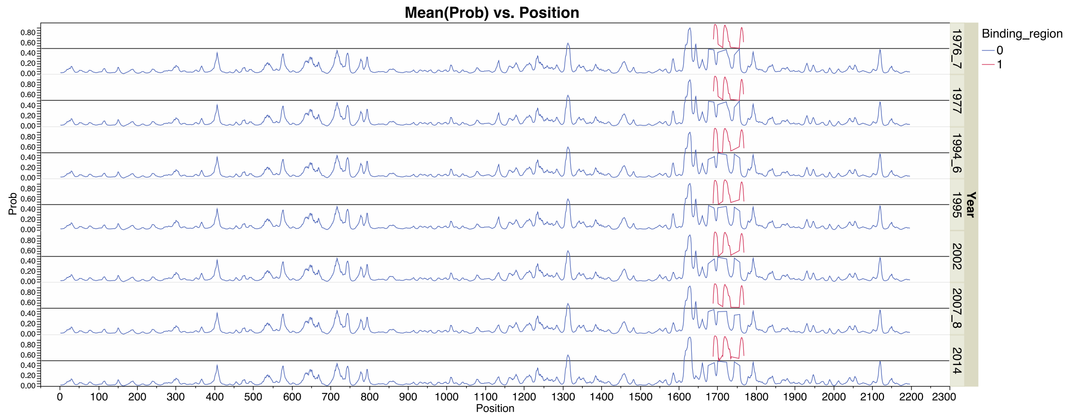

Supplementary Figure 2. Prediction of disordered binding regions. Red lines indicate that the region is predicted to be a binding region, whereas blue curves are indicated that the region is not likely to be involved in binding. Different curves are linked by straight lines. The probability cut off is 0.5. Predictions are made for consensus sequences from each outbreak, using ANCHOR[18]. Predictions are shown for: A. the GP protein; B. NP protein and C. L-polymerase.
